# Emergence of Plasmid-Borne *dfrA14* Trimethoprim Resistance Gene in *Shigella sonnei*

**DOI:** 10.3389/fcimb.2016.00077

**Published:** 2016-07-20

**Authors:** Alfonso Miranda, Bárbara Ávila, Patricia Díaz, Lina Rivas, Karen Bravo, Javier Astudillo, Constanza Bueno, María T. Ulloa, Germán Hermosilla, Felipe Del Canto, Juan C. Salazar, Cecilia S. Toro

**Affiliations:** Programa de Microbiología y Micología, Facultad de Medicina, Instituto de Ciencias Biomédicas, Universidad de ChileSantiago, Chile

**Keywords:** *Shigella sonnei*, trimethoprim resistance genes, *dfrA14*, molecular epidemiology, MDR plasmid, antimicrobial resistance mechanisms

## Abstract

The most common mechanism of trimethoprim (TMP)-resistance is the acquisition of dihydrofolate reductase enzyme resistant to this drug. Previous molecular characterization of TMP-genes resistance in Chilean isolates of *Shigella sonnei* searching for *dfrA1* and *dfrA8*, showed solely the presence of *dfrA8* (formerly *dhfrIIIc*). However, these genetic markers were absent in *S. sonnei* strains further isolated during an outbreak in 2009. To identify the TMP-resistance gene in these strains, a genomic DNA library from a TMP-resistant (TMP^R^) *S. sonnei* representative strain for the outbreak was used to clone, select and identify a TMP-resistance marker. The TMP^R^ clone was sequenced by primer walking, identifying the presence of the *dfrA14* gene in the *sul2-strA'-dfrA14-‘strA-strB* gene arrangement, harbored in a native 6779-bp plasmid. The same plasmid was isolated by transforming with a ~4.2 MDa plasmid extracted from several TMP^R^ *S. sonnei* strains into *Escherichia coli*. This plasmid, named pABC-3, was present only in *dfrA14*-positive strains and was homologous to a previously described pCERC-1, but different due to the absence of an 11-bp repetitive unit. The distribution of *dfrA1, dfrA8*, and *dfrA14* TMP-resistance genes was determined in 126 TMP^R^ *S. sonnei* isolates. Most of the strains (96%) carried only one of the three TMP-resistance genes assessed. Thus, all strains obtained during the 2009-outbreak harbored only *dfrA14*, whereas, *dfrA8* was the most abundant gene marker before outbreak and, after the outbreak *dfrA1* seems have appeared in circulating strains. According to PFGE, *dfrA14-positive* strains were clustered in a genetically related group including some *dfrA1*- and *dfrA8*-positive strains; meanwhile other genetic group included most of the *dfrA8*-positive strains. This distribution also correlated with the isolation period, showing a dynamics of trimethoprim genetic markers prevalent in Chilean *S. sonnei* strains. To our knowledge, *dfrA14* gene associated to a small non-conjugative plasmid was detected for the first time in *Shigella*. Apparently, the strain causing the outbreak must have been introduced, changing drastically the genetic distribution of trimethoprim resistance in Chilean *S. sonnei* strains.

## Introduction

*Shigella sonnei* has become a major problem of public health due to the increasing multidrug-resistance to antibiotic (MDR) worldwide (Ashkenazi et al., 2003; De Lappe et al., 2003; Seol et al., 2006; Vrints et al., 2009). This pathogen, one of the most frequent etiologic agents of foodborne diseases in industrialized countries, is responsible for shigellosis, an acute enteric disease for which antimicrobial therapy is usually recommended to manage infection and reduce fecal excretion of the bacterium to prevent further dissemination. Consequently, a dramatic increase in the rate of resistance to commonly used drugs is observed. One example is the resistance to sulfamethoxazole and trimethoprim (TMP) that has increased since they were introduced as antimicrobial therapy (Huovinen et al., 1995; Huovinen, 1997), especially in *Escherichia coli* and *Shigella*.

Molecular mechanisms of cotrimoxazole (sulfamethoxazole/trimethoprim) resistance could be explained by resistance to trimethoprim and/or to sulfonamides. Specifically for TMP-resistance, several mechanisms have been described (Huovinen et al., 1995; Huovinen, 1997, 2001); however, the most common mechanism is the acquisition of dihydrofolate reductase (DFR) enzyme resistant to this drug. More than 30 different genes are identified encoding TMP-resistant enzymes. They can categorize in two major families based on the length of the N termini of the enzymes and the resistance level they confer: Family A coded by *dfrA* genes and B coded by *dfrB* genes (Recchia and Hall, 1995; Seputiené et al., 2010). The most common *dfr* genes described in *Enterobacteriaceae* are encoded by plasmids, transposons or integrons (Seputiené et al., 2010; Ke et al., 2011; Labar et al., 2012; Cavicchio et al., 2015; Shin et al., 2015). In Chile, between 40 and 60% of the strains isolated from shigellosis cases in the last 5 years corresponds to *S. sonnei*. Noteworthy, most of these isolates are resistant to multiple antimicrobial agents. Surveillance of *in vitro* susceptibility to antimicrobials demonstrated that *S. sonnei* have evolved to MDR isolates (Marcoleta et al., 2013). Particularly, it is notable that resistance to cotrimoxazole increased from 50 to 100%, in strains isolated since 1995 to 2009, year in which an outbreak occurred. The outbreak (739 cases) mainly affected children under 10 years from Región Metropolitana, and the source of infection was not clearly established (Instituto de Salud Pública, 2009).

Previous molecular characterization of TMP-gene resistance in Chilean isolates of *S. sonnei*, searching for *dfrA1* and *dfrA8*, showed solely the presence of *dfrA*8 (formerly *dhfr*IIIc) (White and Rawlinson, 2001), linked to a conjugative plasmid harboring also the *bla*TEM gene (Toro et al., 2005). However, *S. sonnei* strains isolated during the outbreak in 2009 had been negative in detection of *dfrA1* and *dfrA8* TMP-resistance genetic markers. Therefore, in this study we identified and characterized the genetic determinants of TMP-resistance present in *S. sonnei* strains isolated during this outbreak and the distribution of those markers in strains isolated from 1995 to 2013.

## Materials and methods

### Bacterial strains and culture conditions

One hundred twenty-six TMP^R^ *S. sonnei* strains obtained in Chile from 1995 to 2013, from stool samples of patients suffering acute diarrhea were studied. These strains were identified by conventional and automated biochemical methods (VITEK-2, Biomérieux), and serotyped by agglutination with type-specific antisera (Denka-Seiken, Tokyo, Japan). In addition, 51 foreign *S. sonnei* strains, kindly provided by Dr. FX. Weill from the Institut Pasteur collection, were included in this study (Table 1). *E. coli* DH5α nalidixic acid resistant strain (NAL^R^) was used as the recipient in transformation and conjugation experiments. All bacterial strains were routinely cultured at 37°C on LB broth or agar, or trypticase soy, supplemented with ampicillin (AMP) 100 mg L^−1^, NAL or TMP 30 mg L^−1^ when it was required. *E. coli* V517 and *E. coli* 39R861 were used as plasmid size standards.

**Table 1:** **Antimicrobial susceptibility characterization of 51 ***Shigella sonnei*** strains isolated since 1943 to 2006 from different origins worldwide**.

**Name ID**	**Country**	**Region**	**Year**	**NAL**	**AMP**	**STR**	**SUL**	**TMP**	**SXT**	**CHL**	**TET**
4374	France	Europe	1974	S	S	S	R	S	S	S	R
5827	Madagascar	East Africa and Madagascar	2000	S	S	S	R	R	R	S	S
74369	France	Europe	2007	S	R	R	R	R	R	S	S
54216	Sweden	Europe	1946	S	S	S	S	S	S	S	S
54181	Sweden	Europe	1945	S	S	S	S	S	S	S	S
476	France	Europe	1976	S	S	S	S	S	S	S	S
54213	France	Europe	1945	S	S	S	S	S	S	S	S
1567	Senegal	North/West/ Central Africa	1967	S	S	S	S	S	S	S	S
1263	France	Europe	1963	S	S	S	S	S	S	S	S
4474	France	Europe	1974	S	S	S	S	S	S	S	S
1460	France	Europe	1960	S	S	S	S	S	S	S	S
1461	France	Europe	1961	S	S	S	S	S	S	S	S
1167	France	Europe	1967	S	S	S	S	S	S	S	S
1274	France	Europe	1974	S	S	S	S	S	S	S	S
1265	France	Europe	1965	S	S	S	S	S	S	S	S
259	France	Europe	1959	S	S	S	S	S	S	S	S
1761	France	Europe	1961	S	S	S	S	S	S	S	S
1267	France	Europe	1967	S	S	S	S	S	S	S	S
1173	France	Europe	1973	S	S	S	S	S	S	S	S
1166	France	Europe	1966	S	S	S	S	S	S	S	R
54179	Sweden	Europe	1944	S	S	S	S	S	S	S	S
54190	Sweden	Europe	1945	S	S	S	S	S	S	S	S
54185	Denmark	Europe	1945	S	S	S	S	S	S	S	S
54178	Sweden	Europe	1945	S	S	S	S	S	S	S	S
373	Cameroon	North/West/Central Africa	1973	S	S	S	S	S	S	S	S
658	France	Europe	1958	S	S	S	R	S	S	S	S
9810267	Madagascar	East Africa and Madagascar	1998	S	S	S	S	S	S	S	S
41191	Tanzania	East Africa and Madagascar	2004	S	S	S	S	S	S	S	S
54228	Sweden	Europe	1947	S	S	S	S	S	S	S	S
66470	Haiti	Caribbean/Central America	2006	S	R	R	R	R	R	S	R
2574	France	Europe	1974	S	S	R	R	S	S	S	S
998911	France	Europe	1999	S	R	S	S	S	S	S	S
55623	Morocco	North/West/Central Africa	2005	S	S	S	S	S	S	S	S
273	Senegal	North/West/Central Africa	1973	S	S	S	S	S	S	S	S
2073	France	Europe	1973	S	S	S	S	S	S	S	S
8883	France	Europe	1983	S	S	S	S	S	S	S	S
54210	Sweden	Europe	1943	S	S	S	S	S	S	S	S
54184	Sweden	Europe	1945	S	S	S	S	S	S	S	S
989560	Madagascar	East Africa and Madagascar	1998	S	R	R	S	S	S	R	R
66396	France	Europe	2006	S	S	S	S	R	S	S	S
65387	Morocco	North/West/Central Africa	2006	S	S	R	R	R	R	S	R
65179	Senegal	North/West/Central Africa	2006	R	S	R	R	R	R	S	R
2225	French Guiana	South America	2000	S	S	R	R	R	R	S	R
988743	French Guiana	South America	1998	S	S	S	S	S	S	S	S
970044	New Caledonia	Pacific	1997	S	S	R	S	R	S	S	S
31382	Israel	Middle East	2003	S	R	R	R	S	S	R	R
62542	Burkina Faso	North/West/Central Africa	2006	S	S	R	R	R	R	S	R
36224	Senegal	North/West/Central Africa	2003	S	S	R	R	R	R	S	R
65623	Morocco	North/West/Central Africa	2006	S	S	R	R	R	R	S	R
32222	Cuba	Caribbean/Central America	2003	S	S	R	R	R	R	S	R
60108	French Guiana	South America	2006	S	S	R	R	R	R	S	R

*Susceptibility to the antibiotics is expressed as susceptible (S) or resistant (R) strain*.

### Antimicrobial susceptibility

Antimicrobial susceptibility was determined by disk diffusion and microdilution methods by following Clinical and Laboratory Standards Institute guidelines (Clinical Laboratory Standars Institute, 2013). AMP, 10 μg; NAL, 30 μg; ciprofloxacin (CIP), 5 μg; chloramphenicol (CHL), 30 μg; streptomycin (STR), 10 μg; sulfamethoxazole/trimethoprim (SXT), 23.75/1.25 μg; tetracycline (TET), 30 μg and trimethoprim (TMP), 30 μg, were used for disk diffusion test. The reference strain *E. coli* ATCC 25922 was included as a quality control. For analysis purposes, intermediate and resistant isolates were considered together.

### Construction of *S. sonnei* DNA library

Genomic DNA was obtained from a selected *S. sonnei* TMP^R^ strain, grown overnight in LB media at 37°C, using the kit E.Z.N.A *Bacterial*^®^ *DNA Kit* as described by the provider (Omega Bio-Tek, USA). Further, partially *Hin*dIII-digested DNA was separated in 1% agarose gel and the fragments ranging from 2 to 10 kbp were purified using QIAquick gel extraction kit (Qiagen). Meanwhile, the pUC19 plasmid was digested with *Hin*dIII, dephosphorylated with the thermo labile alkaline phosphatase *FastAP* as described by the vendor (Thermo-Scientific, USA), and gel-purified. Both, the genomic DNA fragments and the digested plasmid were incubated with T4 DNA ligase (Thermo-Scientific, USA) for 24 h at 4°C in a ratio of 3:1 (insert: vector). The ligation mixture was transformed into *E. coli* DH5α cells, and recombinants were recovered overnight at 37°C in presence of TMP 50 mg L^−1^. The recombinant plasmid obtained (pCLON3) was purified from the isolated TMP^R^ *E. coli*, using *QIAprep Spin Miniprep Kit* (QIAGEN) and sequenced (Macrogen, Korea). The 6779-bp fragment cloned was released from the recombinant plasmid pCLON3 by *Hin*dIII digestion; then gel-purified, self-ligated and transformed into *E. coli* DH5α. Finally, *E. coli* transformants selected in TMP were confirmed to carry the reconstituted native plasmid, named pABC-3. The TMP^R^ genetic marker harbored in this plasmid was *dfrA14*.

### Detection of trimethoprim resistance markers

DNA templates were obtained from individual colonies by bacterial lysis or by purifying total genomic DNA. Primers used are indicated in Table 2. PCR reactions for *dfrA1, dfrA8, dfrA14* were performed as follow: an initial 2 min denaturation cycle at 95°C followed by 30 cycles at 95°C for 1 min, 56°C for 30 s, and 72°C for 1 min for *dfr* primers respectively, with a final extension at 72°C for 10 min. PCR products were analyzed by electrophoresis in 2% agarose gels and stained with ethidium bromide.

**Table 2:** **PCR primers used in this study**.

**Primers**	**5′-Sequence-3′**	**Tm**	**Expected Product size**	**Reference**
dfrA1-Fw	GGAGTGCCAAAGGTGAACAGC	59	367 bp	Toro et al., 2005
dfrA1-Rv	GAGGCGAAGTCTTGGGTAAAAAC	56		Toro et al., 2005
dfrA8-Fw	GAGCTTCCGGGTGTTCGTGAC	60	247 bp	Toro et al., 2005
dfrA8-Rv	CTTCCATGCCATTCTGCTCGTAGT	59		Toro et al., 2005
dfrA14-Fw	TTAACCCAGGATGAGAACCT	56	510 bp	This work
dfrA14-Rv	CGATTGCATAGCTTTGTTAA	54		This work
pCLON3-Fw2	ATAGCCGATCAAATGATGAG	52	^*^	This work
pCLON3-Rv2	TAACGAATTCTTGCGGTTTC	55	^*^	This work
pCLON3-Fw3	CACTCCCACTCTTACATTGT	52	^*^	This work
pCLON3-Rv3	GGTTTAACCGTAATCAACAG	52	^*^	This work
pCLON3-Fw4	TAAAACAGGCAACAAACCAC	56	^*^	This work
pCLON3-Rv4	GCGAATATGACCTTTTTGAT	54	^*^	This work
strA-Fw^a^	TGACTGGTTGCCTGTCAGA /	60	1000 bp	This work
rcr2-R^b^	/ GCTGGAGCATGGCTTTCTAC	60	1993 bp	This work

* *used to sequence by primer walking*.

a *reverse primer to amplify a 1000-bp fragment is dfrA14-Rv*.

b *forward primer to amplify a 1993-bp fragment is dfrA14-Fw*.

### Purification and isolation of native plasmid containing the *dfrA14* gene

The native plasmids were obtained from a representative *S. sonnei* strain using E.Z.N.A. plasmid DNA mini kit II (OMEGA Bio-Tek) or by alkaline extraction, separated in a 1% agarose gel electrophoresis, and visualized with ethidium bromide. Two plasmids of ~4.2 MDa were purified using QIAquick gel extraction kit (Qiagen). Chemically competent *E. coli* DH5α (Inoue et al., 1990) were transformed with 250 ng of the plasmid gel-isolated and selected in presence of TMP 30 mg L^−1^. Finally, plasmid extracted from the TMP^R^ transformant bacteria, was sequenced and compared with the reconstituted plasmid, pABC-3, showing 100% identity. pABC-3 restriction analysis *in silico* was performed using NebCutter software. *Ssp*I was selected for restriction fragment long polymorphism (RFLP) analysis producing 3 bands of 2920, 2343, and 1516 bp. Restriction was made using 5 μg of plasmid and *Ssp*I 1U μg^−1^ in 20 μL of mix reaction during 2 h at 37°C.

### Conjugation analysis

Conjugations were carried out with 10 TMP^R^- *S. sonnei* strains using NAL^R^- *E. coli* DH5α as recipient. The overnight culture of each purified donor and recipient cells were diluted 100-fold in 2 mL of fresh LB broth and incubated at 37°C until OD_600_ reached about 0.6. Then 0.2 mL each of the donor and recipient cultures was mixed. After incubation at 37°C without shaking for 3–4 h, the mixture was plated on LB agar supplemented with TMP 30 mg L^−1^ and NAL 30 mg L^−1^. Plasmid extraction and PCR reactions for the *dfr*A*14* gene were applied to confirm the transconjugants that acquired plasmids and resistance genetic marker. Changes of the susceptibility to antimicrobial agents were measured by the disk diffusion method as described above.

### Sequence analysis

Comparative analysis of nucleotide sequences was performed using BLAST at the National Center for Biotechnology Information (NCBI) (site www.ncbi.nlm.nih.gov/BLAST/). The pABC-3 plasmid sequence was deposited at the GenBank database under accession number KT988306. Graphic representation of the loci alignment was performed using EasyFig v2.1 (Sullivan et al., 2011), with the blastn algorithm. Plasmid sequences incorporated in this comparison were: *Klebsiella pneumoniae* pKDO1 (GenBank accession number NC_019389.1), *E. coli* pPGRT46 (KM023153.1), *E. coli* pSTOJO1 (AJ313522.1), *Yersinia ruckeri* pYR1521 (NG_041026.1), *E. coli* pCERC1 (NC_019070.1), *S. sonnei* pKKTET7 (NC_008439.1), *S. sonnei* pSS4 (AF534183.1), *Salmonella* Typhimurium pSRC15 (NC_013104.1), *Shigella flexneri* pSFxv_3 (CP001386.1), *E. coli* pCN061p3 (CP006639.1), *Pasteurella multocida* pVM111 (NG_035903.1). More information about these plasmids is detailed in the Supplementary Table.

### Pulsed field gel electrophoresis (PFGE)

Genomic DNA from the selected strains was included into agarose plugs and digested with endonuclease *Xba*I (Thermos Scientific). According to PulseNet protocol (CDC), the macrorestriction fragments were separated by pulsed-field gel electrophoresis on a CHEF-DRIII Chiller system (Bio-Rad Laboratories, Richmond, CA), in 1% agarose gel using 0.5X TBE buffer at 6 V/cm and 14°C, with ramped pulse times of 2.2 to 54.2 s for 21 h. *Salmonella enterica* serovar Braenderup was included as molecular size standard. The DNA band profiles were analyzed with GelCompar software (version 3.0; Applied Maths, Sint-Martens-Latem, Belgium). The cluster analysis and generation of dendrograms was performed using UPGMA with a 1.5% band tolerance. The similarity between DNA profiles was determined using Dice's correlation coefficient. We defined arbitrarily pulsotypes and pulsogroups with similarities of >91.1% and 75%, respectively.

## Results

### Identification of *dfrA14* TMP-resistance genetic marker in *S. sonnei* strains

**Figure 1:**
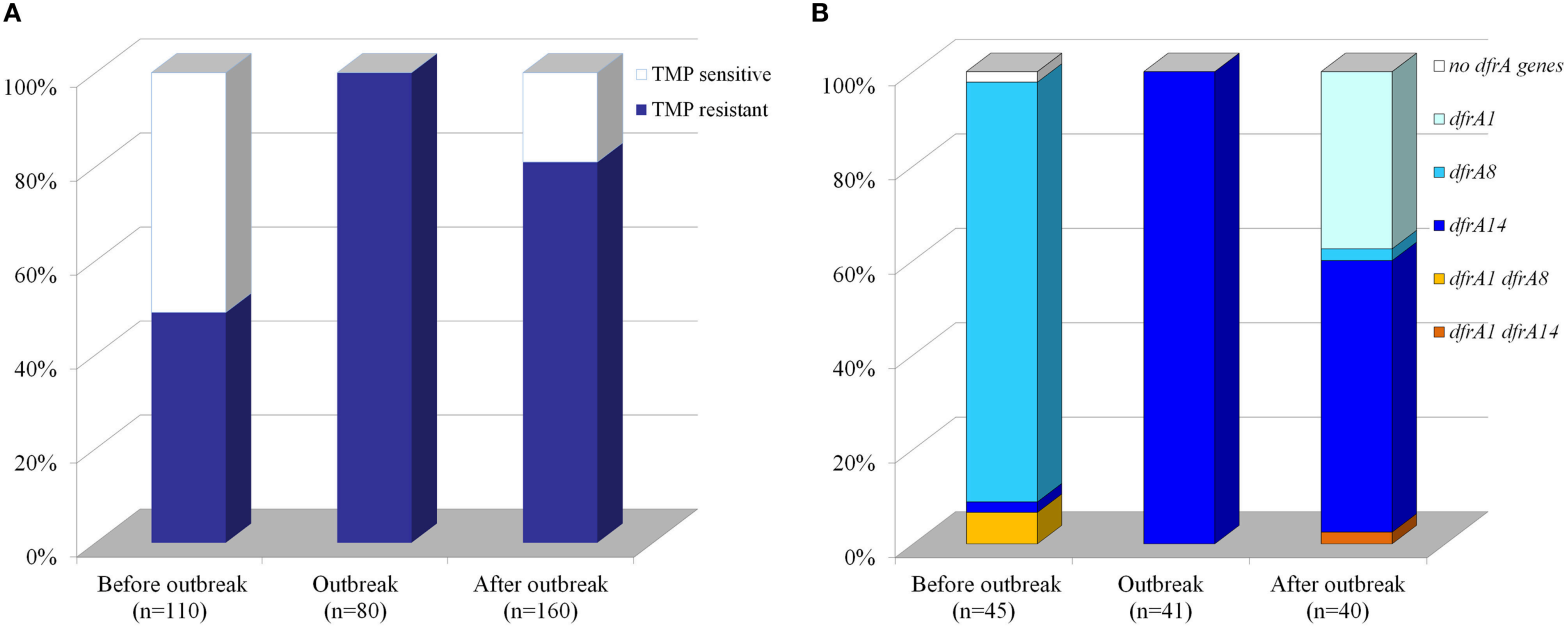
**Distribution of trimethoprim-resistant strains and trimethoprim-genetic markers among Chilean ***Shigella sonnei*** isolates. (A)** Percentage of TMP-susceptible and–resistant strains. Susceptibility to TMP was measured by disk diffusion in a total of 350 strains isolated before, during or after the 2008–2009 outbreak. A total of 264 strains were TMP^R^ including the 80 strains obtained during the outbreak. **(B)** Percentage of strains harboring TMP-resistance genetic markers. Presence of *dfrA1, dfrA8*, and *dfrA14* was assessed by PCR in a smaller group (126 strains) randomly selected from TMP^R^ *S. sonnei* strains.

Resistance to TMP has been increasing rapidly worldwide, since this antibiotic was introduced. Chilean *S. sonnei* strains isolated from 1995 to 2013 also displayed increased level of TMP-resistance as measured by disk diffusion, from 49 up to 81% and exceptionally 100% during the outbreak in the summer of 2008–2009 (Figure 1A). Seventy-five percent, 264 out of the total amount of 350 *S. sonnei* strains, were TMP^R^. To characterize the molecular determinants of TMP-resistance in these Chilean *S. sonnei* strains, we randomly chose 126 TMP^R^ strains obtained from different periods, divided as 3 groups: 45 strains before the outbreak (isolated since 1995–1997 and 2004–2006 period), the 41 strains during the outbreak (2008–2009), and 40 strains isolated after the outbreak (2010–2013). Previously, we demonstrated that *S. sonnei* strains isolated in 1995 harbored solely *dfrA8* gene (Toro et al., 2005). Detection of *dfrA1* and *dfrA8* by PCR was negative in the 41 *S. sonnei* strains collected during the 2008-2009 outbreak.

To identify the TMP-resistance gene in these strains, a DNA library was done in pUC19 (Amp^R^) using *Hin*dIII-digested genomic DNA from the TMP^R^ *S. sonnei* C8225 as representative strain for the outbreak and *E. coli* DH5α as host. Recombinant plasmids from TMP^R^ clones were selected, purified, and transformed into a new *E. coli* DH5α, isolating the TMP-genetic marker in the plasmid named pCLON3. The *dfrA14* gene was identified as the TMP-resistance marker by sequencing the insert of this recombinant plasmid and comparing with available data in the GenBank database.

### Distribution of TMP-resistance gene markers in *S. sonnei* strains

To determine the presence of *dfrA14* gene in Chilean *S. sonnei* strains, specific primers for this marker were designed (Table 2). Thus, only 1 out 45 strains isolated before the outbreak harbored *dfrA14*, meanwhile 100% of strains belonged to the outbreak displayed only the *dfrA14* TMP-genetic marker and 24 out 40 isolated after the outbreak carried this gene (Figure 1B). Most of the strains isolated before the outbreak (96%) displayed the *dfrA8* gene marker. In contrast, *dfrA1* was detected in 40% after the outbreak. The *dfrA1*/*dfrA8* combination was detected only in three strains isolated before the outbreak and *dfrA1*/*dfrA14* in one strain isolated after the outbreak. The *dfrA8*/*dfrA14* combination was not present in this Chilean group of strains (Figure 1B).

Considering the 126 strains, most of them (96%) carried one of the three TMP-resistance gene assessed; only one strain (0.8%), isolated before the outbreak was negative for the three genes, and just 4 strains (3%) displayed two resistance genes. Fifty-two percent of all strains harbored only *dfrA14*, whereas 32% of strains displayed *dfrA8* and 12% *dfrA1*.

### Characterization of the plasmid-borne *dfrA14* gene in *S. sonnei* strains

The recombinant plasmid harboring the *dfrA14* TMP-marker (pCLON3) sequenced by primer walking, was bioinformatically analyzed and sequences revealed an insert of a 6779-bp fragment harboring the *dfrA14* cassette in the *sul2-strA'-dfrA14-‘strA-strB* arrangement. Alignment of the complete 6779-bp fragment showed most likely identity with the pCERC-1 plasmid (Anantham and Hall, 2012; accession number JN012467.1). Only one specific difference was detected compared to pCERC-1 plasmid: a deletion of an 11-bp short repeat at orf3.

**Figure 2:**
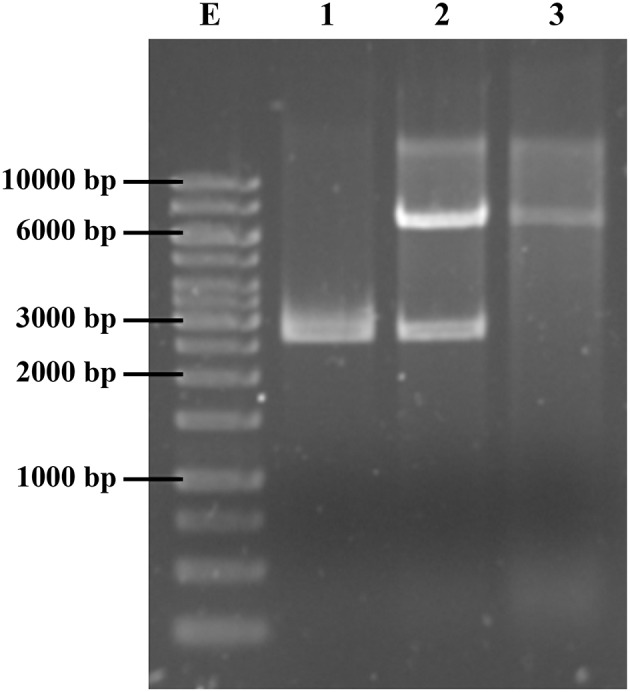
**Isolation of plasmid pABC-3, harboring the ***dfrA14*** gene**. Lane 1, pUC19 digested with *Hin*dIII. Lane 2, pCLON3, recombinant pUC19 harboring a ~7 kb fragment TMP-resistance marker, digested with *Hin*dIII. Lane 3, the native pABC-3 plasmid reconstituted by self-ligation and recovered from DH5α TMP^R^ transformants, digested with *Hin*dIII.

According to this analysis, the cloned sequence corresponded to a whole native plasmid. To validate this finding, the insert was released from the recombinant plasmid pCLON3 by *Hin*dIII digestion (Figure 2, lane 2); then, the 6779-bp fragment was gel-purified, self-ligated and transformed into *E. coli* DH5α. Finally, transformants selected in TMP were confirmed to carry the reconstituted native plasmid with the TMP^R^ genetic marker, *dfrA14* (Figure 2, lane 3). This plasmid was named pABC-3 (GenBank accession number KT988306). Wild type and transformants strains were checked for their antimicrobial susceptibility profile to 8 antibiotics. *E. coli* transformant strains with pCLON3 and pABC-3 were resistant to NAL, SXT, and TMP but susceptible to tetracycline and streptomycin, measured by disk diffusion, indicating that *sul2* present in the cloned plasmid is functional and the *str* genes are mutated by *dfrA14* insertion. pCLON3 coded additionally the Amp-resistance harbored in the pUC19 vector. MIC to TMP was measured in *S. sonnei* C8225, DH5α/pCLON3 and DH5α/pABC-3. All of them raised the MIC over 1000 mg L^−1^, as expected for a *dfr* type A coded enzyme as *dfrA14*.

**Figure 3:**
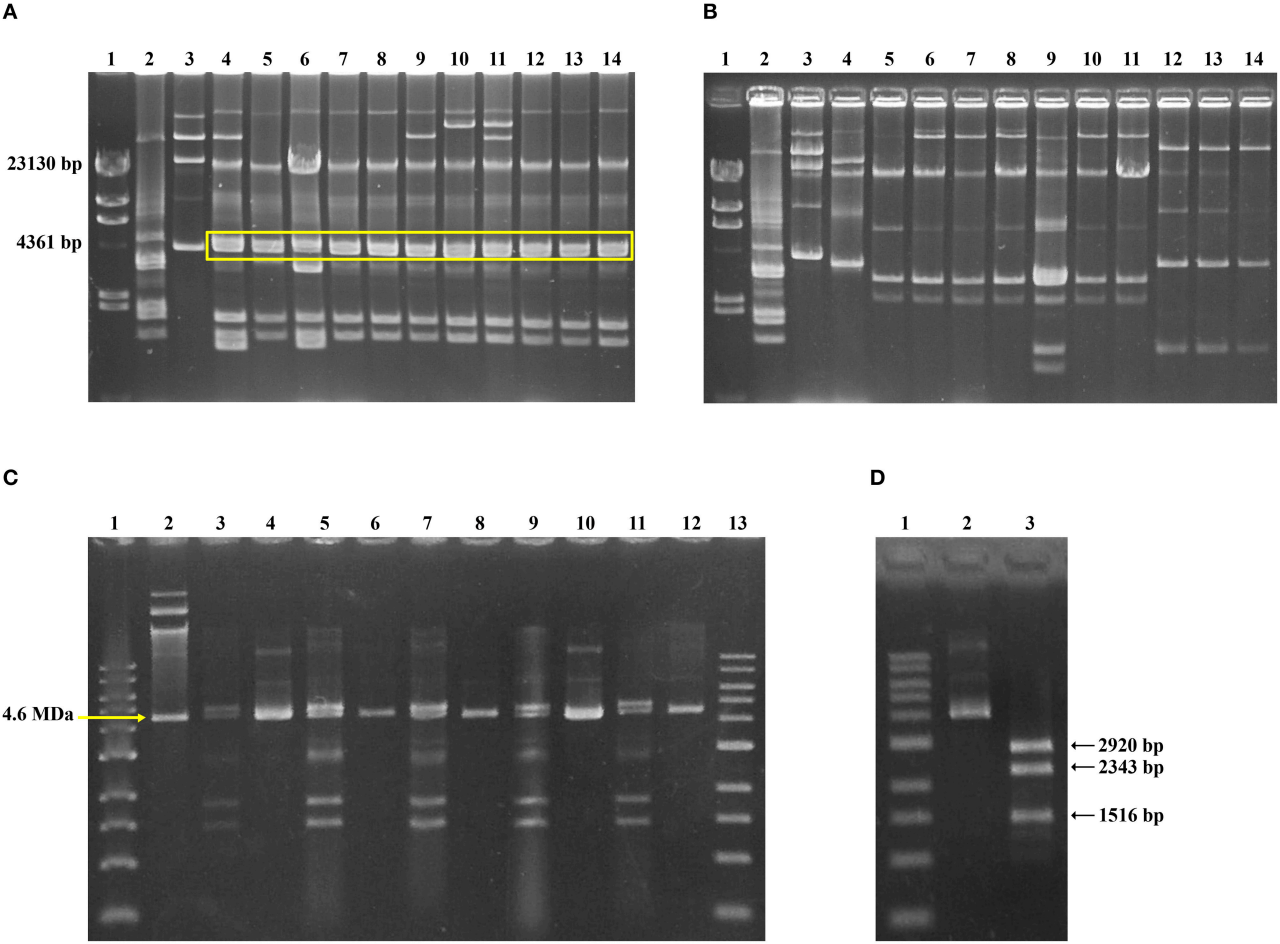
**Characterization of pABC-3 plasmid in Chilean TMP-resistant ***Shigella sonnei*** strains**. Plasmid profiles of representative TMP^R^ *S. sonnei* strains isolated during the outbreak (**A**, lane 4 – 14) and before the outbreak (**B**, lane 4 – 14), obtained by alkaline extraction and separation in 1% agarose gel electrophoresis. Molecular size markers in **(A,B)**: lane 1, Lambda *Hin*dIII DNA marker; lane 2, *E. coli* V517; lane 3, *E. coli* 39R861. A duplex band migrating near to 4.2 MDa is highlighted by a yellow line in panel A, which is absent in **(B)**. **(C)** Involvement of pABC-3 plasmid in TMP-resistance. Plasmid profiles from representative TMP^R^ *S. sonnei* strains next to the corresponding TMP^R^ *E. coli* DH5α transformant are shown. Lane 1 and 13, 1 kb molecular size marker (*New England Biolabs*^®^); lane 2, *E. coli* 39R861; lane 3 – 4, *S. sonnei* strain c0027 and the respective TMP^R^*E. coli* DH5α transformant; lane 5 – 6, *S. sonnei* strain c0700 and the TMP^R^ transformant; lane 7 – 8, *S. sonnei* strain c0719 and the TMP^R^ transformant; lane 9 – 10, *S. sonnei* strain c8072 and the TMP^R^ transformant; lane 11 – 12, *S. sonnei* strain c8205 and the TMP^R^ transformant. **(D)** A representative *Ssp*I digestion pattern of pABC-3, in concordance with the *in silico* predicted size. Lane 1, 1 kb molecular size marker (*New England Biolabs*^®^); lane 2, pABC-3 plasmid undigested; lane 3, pABC-3 plasmid digested with *Ssp*I.

In parallel, the plasmid electrophoretic profiles for *S. sonnei* TMP^R^ *dfrA14*-positive strains isolated in 2008-2009 was analyzed. As Figure 3A shows, a pattern including a double band of ~4.2 MDa, was found in all of them. Size estimation of plasmids was done by comparison with *E. coli* V517 and *E. coli* 39R861 standards plasmids. On the other hand, the double band was absent in *S. sonnei* TMP^R^ strains isolated before the outbreak (Figure 3B). To determine whether one of those plasmids harbor the *dfrA14* gene, double band gel-isolated of several *dfrA14*–positive *S. sonnei* strains belonged to the outbreak was used to transform *E. coli* DH5α and to select TMP-resistance. By this way, it was possible to isolate one plasmid, whose size was similar to the smaller band (Figure 3C). The detection of *dfrA14* gene was positive only for TMP^R^ transformants. RFLP of this plasmid was conducted using plasmid DNA extracted from 10 different *E. coli* transformant strains, using *Ssp*I restriction enzyme; thus, it was verified that isolated plasmids harbored the same restriction pattern, as expected for pABC-3 (Figure 3D), suggesting the presence of the same plasmid. Complete sequences of both TMP^R^ plasmids, the one isolated by direct transformation and the other one reconstituted from the TMP^R^ clone, pABC-3, were compared and it was confirmed that they were identical.

To link the *dfrA14* gene to the presence of the *sul2-strA'-dfrA14-‘strA-strB* arrangement and the 6779-bp plasmid in the rest of the strains, two overlapping PCR reactions were designed to detect the neighborhood of *dfrA14* gene (Table 2). All of the TMP^R^ strains were positive for both reactions, indicating the *dfrA14* marker was harbored in this plasmid (data not shown).

In addition, the presence of *dfrA14* was evaluated in 51 foreign *S. sonnei* strains, isolated since 1943 to 2006 from different origins worldwide (Table 1). Thirteen were TMP^R^, and they did not harbor this marker, however, 10 of them display only *dfrA1*.

Conjugation experiments were done to determine whether the native pABC-3 plasmid was transferable. However, no transconjugants cells were obtained in TMP when *dfrA14*–positive *S. sonnei* strains were used as donors. As control, *dfrA*8 and *bla*TEM could be conjugated in the same experimental conditions (Toro et al., 2005). Consequently, no mobilization genes were identified in the sequence of pABC-3. Further *in silico* analysis detecting incompatibility group based on PCR-based replicon typing (PBRT) showed no similarity with the 18 major incompatibility (Inc) groups of *Enterobacteriaceae* species (Carattoli et al., 2005). Moreover, using plasmid finder to identify replication origins (http://www.genomicepidemiology.org), using a threshold of 80% we detected 89% identity only with *ColRNAI* (accession number DQ298019.1), suggesting the presence of an undescribed variant plasmid (Carattoli et al., 2014).

### Clonal analysis of TMP-resistant *S. sonnei* strains

**Figure 4:**
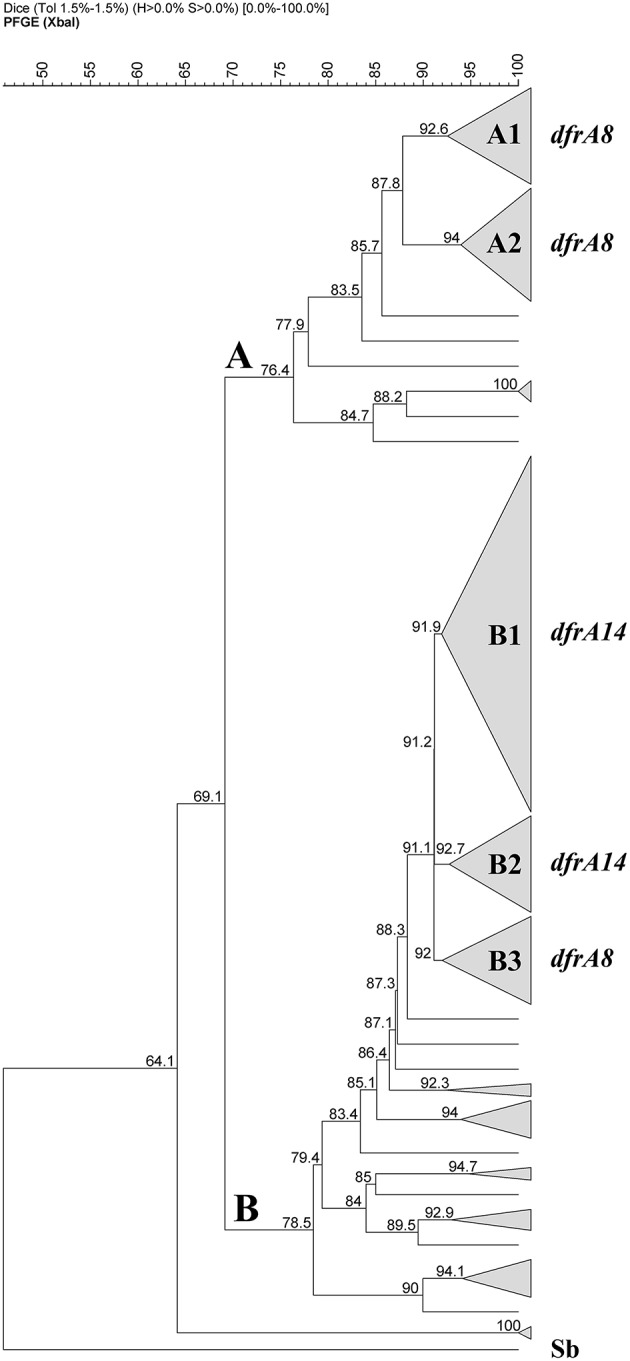
**Dendrogram based on Dice coefficients of similarity for 126 TMP-resistant ***Shigella sonnei*** strains**. The dendrogram was constructed by UPGMA (GelCompar II). Figure shows two pulsogroups **(A,B)** with a similarity of 69.1%. Twenty-three pulsotypes were defined with a similarity >91%. Within the pulsogroup A, most of the strains were grouped in A1 and A2 pulsotypes, both containing *dfrA8* as TMP-resistance marker. Within the pulsogroup B, most of the strains were clustered in three pulsotypes, B1 and B2 include the *dfrA14*-positive strains and B3 *dfrA8*-positive strains isolated before the outbreak. Sb, strain of *Salmonella* serotype Braenderup, digested with *Xba*I as a size marker.

PFGE was performed for all the 126 TMP^R^ *S. sonnei* strains using *Xba*I digestion. Figure 4 shows two pulsogroups: A is the minor group included 34 strains, 32 of them belonged to the 45 strains isolated before the outbreak (71%); pulsogroup B is a more heterogenous group (*n* = 92 strains), including most of the strains isolated during and after the outbreak (97%, *n* = 79/81). Considering the distribution of *dfrA* genes, within the pulsogroup A, 32 of them were *dfrA8*-positive strains and separated in two pulsotypes A1 and A2. In the pulsogroup B, the most frequent profiles are clustered in three pulsotypes. The pulsotype B1 contained 43 strains, from which 40 (93%) harbored the *dfrA14* gene; the B2 grouped 12 strains, 11 (92%) harbored the *dfrA14* gene. Noteworthy, the pulsotype B3 (11 strains), contained the only strain negative for the three TMP-resistance genes studied, 6 *dfrA8*-positive strains isolated before the outbreak, 3 *dfrA14*-positive strains isolated after the outbreak and one *dfra1*-positive strain isolated after the outbreak. From the 26 remaining strains from pulsogroup B, most of them presented individual pulsotypes and contained *dfrA1* or *dfrA14* genes.

Together, these results shows a drastic change of the genetic distribution of trimethoprim resistance in Chilean *S. sonnei* strains.

## Discussion

The high increase of TMP-resistance is a worldwide event especially in Gram-negative bacteria (Huovinen et al., 1995; Huovinen, 1997, 2001). In this study, we sought to identify TMP-resistance genes in *S. sonnei* strains that lacked *dfrA1* and *dfrA8*, the previously described *dfr* genes (Toro et al., 2005).

**Figure 5:**
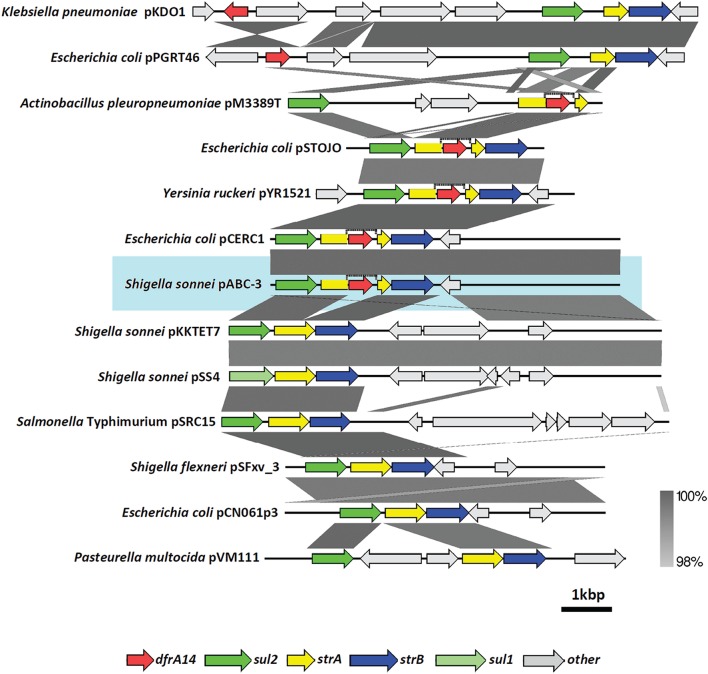
**Comparison of different plasmids harboring ***dfrA14*** gene**. Alignment of *Shigella sonnei* pABC-3 with other antibiotic resistance gene clusters contained in plasmids of Gram negative bacteria, which harbor *dfrA14* and/or the *sul2-strA-strB* arrangement. *dfrA14* gene is represented by the red arrow. The graphic was built with Easyfig using the blastn algorithm. Vertical bar at the right bottom indicates the threshold of identity percentages in the alignments. More details about plasmids are described in Supplementary Table.

Firstly, characterizing TMP^R^ *S. sonnei* strains belonged to the 2009-oubreak, we cloned the TMP-resistance genetic determinant identified as *dfrA14*, formerly named *dhfr*lb (Recchia and Hall, 1995) and harbored in a small plasmid (6779 bp). Sequence of this plasmid was homologous to the pCERC-1, previously found in commensal *E. coli* (Anantham and Hall, 2012). Both plasmids display the same particular arrangement including the *sul2-strA'-dfrA14-‘strA-strB* genes (Figure 5, and Supplementary Table), which has also been described in plasmids isolated from other enterobacteria such as uropathogenic *E. coli* (Ojo et al., 2002) and more recently in a larger plasmid (8.9 kb) from the fish pathogen *Y. ruckeri* (Huang et al., 2014). Interestingly, the *strA'-dfrA14-‘strA* insertion was also reported in the pM3224T plasmid including the same arrangement with *sul2* and *strB* cassettes (6050 bp) and with a different architecture for *strA'-dfrA14-‘strA* and *sul2* genes in the mobilizable pM3389T plasmid (6101 bp). Both of these plasmids are harbored by *Actinobacillus pleuropneumoniae*, a distantly related species belonging to the *Pasteurellaceae* family that causes respiratory infections in pigs (Bossé et al., 2015). Recently, a novel plasmid (83 kb) isolated in commensal *E. coli* carrying *bla*CTX-M-15, *sul2-strA-strB* cluster and *dfrA14* was described; however, the TMP-resistance gene was not inserted in *sul2-strA-strB* arrangement (Fortini et al., 2015).

The antibiotic resistance gene cluster containing *sul2, strA*, and *strB* genes is also widespread among Gram-negative bacteria and this array can display different combinations with other resistance genes different from *dfrA14*, such as *catA3* or *tet* genes, giving rise to new resistance gene clusters (Kehrenberg and Schwarz, 2002; Kehrenberg et al., 2003). In *S. sonnei*, two plasmids similar to pABC-3, pKKTET7 (8401 bp) and pSS4 (8384 bp), have been previously found in strains isolated in Korea, (accession number AF497970-1 and AF534183 respectively). By comparing the pABC-3 (from Chilean strains) with pKKTET7 and pSS4 sequences, it seems that the core is shared among them and the only difference is the presence of *tetR* and *tetA* genes instead of *dfrA14* in the latter two plasmids. This suggests the exchange of antimicrobial resistance cassettes within a common backbone (Figure 5).

On the other hand, *Salmonella* Typhimurium isolated in Australia is an example of bacteria harboring this *sul2-strA-strB* cluster in the pSRC15 plasmid (Figure 5; Yau et al., 2010). In *S. flexneri*, a plasmid named pSFxv_3 (6200 bp) shares almost 100% of sequence identity with pKKTET-7, including the *sul2-strA-strB* region. However, pSFxv_3 did not display *tet* nor *dfr* markers (Ye et al., 2010). Another example in *S. flexneri* is the 4.3-MDa plasmid harboring *sul2* gene but not *dfr* gene markers, suggesting that *sul2-strA*-*strB* gene cluster is present (Iqbal et al., 2014). This plasmid isolated in samples from Bangladesh was not sequenced but it seems to be the same as pSFxv_3. Thus, to our knowledge, the *sul2-strA'-dfrA14-‘strA-strB* cluster found in the *S. sonnei* pABC-3 is described for the first time in *Shigella* (Figure 5).

Earlier reports established that *Shigella* TMP-resistance was mediated by transposable genetic elements inserted in a conjugative, multiple antibiotic-resistance plasmid (Tonin and Grant, 1987). Later, the *dfrA14* gene was described as the dominant gene among TMP^R^-plasmids isolated from uropathogenic bacteria in Scotland. This genetic determinant was sequenced and found to be present within a Tn7-like structure (Young and Hillyear, 1994; Young et al., 1994). These features were not similar to the *dfrA14* harbored by pABC-3, which is a small plasmid without transposon-like elements.

Antimicrobial resistance phenotype in enterobacteria is often associated with the presence of gene cassettes harbored in integrons. Thus, most of the reports have described TMP-resistance linked to class 1 or class 2 integrons. In this scenario, *dfrA1, dfrA12, dfrA15*, and *dfr17* cassettes are the principal genetic marker displayed at integrons in enterobacteria (Pan et al., 2006; Dubois et al., 2007; Gassama-Sow et al., 2010; Ke et al., 2011; Zhu et al., 2011; Shin et al., 2015). Although the majority of *dfr* genes seem to be coded within integrons, just some clusters carrying *dfr*A*14* are associated to class 1 or 2 integrons (Kadlec and Schwarz, 2008; Wei et al., 2014). Bioinformatic analysis from this work demonstrated that *dfrA14* is harbored in pABC-3 without integron-like elements (data not shown). The presence of *dfrA1* and *dfrA8* genes coded in integrons has being analyzed.

Recently, *dfrA14* was found for the first time in *S. flexneri* strains by whole genome sequencing. Integration of the genetic information with geographical and temporal data showed that this gene could be detected since 1990; however, no information about the genetic localization or genetic context is provided (Connor et al., 2015).

Interestingly, comparing the *orf3* sequence described in pCERC-1 among *Shigella* plasmids, we detected a variable region of a 11-bp unit (GATGTAAAAGT) repeated five times in pCERC-1, pKKTET7 and pSS046; however, pSFxv_3 and pSS4 displayed only three out these five repetitions. pABC-3, described in this work, had four repetitions, suggesting that this region may discriminate different plasmids.

According to PFGE, all *dfrA14*-positive strains were clustered in pulsogroup B, grouping 93% of them in pulsotypes B1 and B2, isolated during and after the outbreak. This temporal coincidence suggests the appearance of strains genetically different during the outbreak, harboring the *dfrA14*-coded plasmid. Apparently, the strains causing the outbreak might have been introduced to the country or they were locally selected by horizontal gene transfer; however, our data do not allow us to discriminate between both hypothesis. Moreover, in neighbor countries have no described isolates similar to Chilean strains. Most of reports from Argentine, Peru, and Brazil described the antibiotic resistance profiles of circulating strains, highlighting in some cases the high level of TMP-resistance; however they do not search for *dfrA14* (Merino et al., 2004; Lluque et al., 2015; Seribelli et al., 2016).

To conclude, to our knowledge, this is the first time that the *dfrA14* TMP-resistance gene has been found in *S. sonnei* isolates linked to a small plasmid, becoming one of the most common in Chilean TMP^R^ *S. sonnei* strains, besides *dfrA1* and *dfrA8* genes. Apparently, the strain causing the outbreak must have been acquired, changing drastically the temporal dynamics of trimethoprim resistance in Chilean *S. sonnei* strains and highlighting the urgency to maintain permanent surveillance of antimicrobial resistance profiles and the molecular mechanisms of resistance to improve both prevention and treatment of shigellosis.

## Author contributions

AM: data acquisition, data analysis, data interpretation; BA: data acquisition, data analysis, data interpretation; PD: data analysis, data interpretation, revising of the manuscript; LR: data acquisition, data analysis; JA: data acquisition, data analysis, data interpretation; KB: data acquisition, data analysis, data interpretation; CB: data acquisition, data analysis, data interpretation; MU: study design, data interpretation, revising of the manuscript; GH: data interpretation, revising of the manuscript; FC: data analysis, data interpretation, revising of the manuscript; JS: data analysis, data interpretation, writing of the manuscript, revising of the manuscript; CT: study design, data analysis, data interpretation, writing of the manuscript, revising of the manuscript. MU, GH, and CT are principal investigator at the FONDECYT grant that funded this work.

## Funding

This work was supported by Fondo Nacional de Desarrollo Científico y Tecnológico, FONDECYT grant 1130394.

### Conflict of interest statement

The authors declare that the research was conducted in the absence of any commercial or financial relationships that could be construed as a potential conflict of interest.
