# Supplementary material for: Emergence of Plasmid-Borne dfrA14 Trimethoprim Resistance Gene in Shigella sonnei
Source: Front Cell Infect Microbiol. 2016 Jul 20;6:77. doi: 10.3389/fcimb.2016.00077 (PMC4951496; doi:10.3389/fcimb.2016.00077)
Supplement: Supplementary file 1 [file Table1.DOCX]

Supplementary Table: Presence of *dfrA14* and *sul2 strA strB* arrangement in different plasmids

| **Plasmid name** | **Plasmid size (bp)** | **Organism** | **Isolation source** | **Type** | **Country** | **Year isolated** | **Relevant resistance cassettess** | **Accession number** | **Reference** |
| --- | --- | --- | --- | --- | --- | --- | --- | --- | --- |
| pABC-3 | 6779 | *Shigella sonnei* | Human | Enteropathogen | Chile | *2008-2009* | *sul2 strA::dfrA14 strB* | KT988306 | This work |
| pCERC-1 | 6790 | *Escherichia coli* | Human | Commensal | Australia | *2008* | *sul2 strA::dfrA14 strB* | JN012467.1 | Anatham et Hall, 2012 |
| pSTOJO1 | ~ 6800 | *Escherichia coli* | Human | Uropathogen | Nigeria | *2001* | *sul2 strA::dfrA14 strB* | AJ313522.1 | Ojo et al, 2002 |
| pYR1521 | ~ 8900 | *Yersinia ruckeri* | Fish | Pathogen | Germany | *2004-2012* | *sul2 strA::dfrA14 strB* | HG423538.1 | Huang et al, 2014 |
| pM3224T | 6050 | *Actinobacillus pleuropneumoniae* | Pig | Pathogen | England | *2005-2010* | *sul2 strA::dfrA14 strB* | KP197004 | Bossé et al, 2015 |
| pVM111 | ~ 9800 | *Pasteurella multocida* | Turkey | Pathogen | USA, California | *1975* | *sul2 tetR tetH strA strB* | AJ514834 | Kehrenberg et al, 2003 |
| pM3389T | 6101 | *Actinobacillus pleuropneumoniae* | Pig | Pathogen | England | *2005-2010* | *sul2 /// strA:: dfrA14* | KP197005 | Bossé et al, 2015 |
| pKKTET7 | 8401 | *Shigella sonnei* | Human | Enteropathogen | Gyeongbuk Korea | *1998* | *sul2 strA strB* | AF497970.1 | -- |
| pSS4 | 8384 | *Shigella sonnei* | Human | Enteropathogen | Cheju Island Korea | *2000* | *sul2 strA strB* | AF534183.1 | -- |
| pSFxv_3 | 6200 | *Shigella flexneri* | Human | Enteropathogen | China | *2002* | *sul2 strA strB* | CP001386.1 | Ye et al, 2010 |
| PCN061p3 | 6222 | *Escherichia coli* | Pig | Pathogen | China | *2004-2007* | *sul2 strA strB* | CP006639.1 | Liu et al, 2015 |
| pSRC15 | ~ 8700 | *Salmonella* Typhimurium | Human | Pathogen | Australia | *¿2000?* | *sul2 strA strB* | GQ379901.1 | Yau et al, 2010 |
| pB1003 | 5057 | *Pasteurella multocida* | Pig | Pathogen | Spain | *2002-2005* | *sul2 strA strB* | EU360945 | San Millan et al, 2009 |
